# Mining Outcome-relevant Brain Imaging Genetic Associations via Three-way Sparse Canonical Correlation Analysis in Alzheimer’s Disease

**DOI:** 10.1038/srep44272

**Published:** 2017-03-14

**Authors:** Xiaoke Hao, Chanxiu Li, Lei Du, Xiaohui Yao, Jingwen Yan, Shannon L. Risacher, Andrew J. Saykin, Li Shen, Daoqiang Zhang, Alzheimer’s Disease Neuroimaging Initiative, Michael W. Weiner, Paul Aisen, Ronald Petersen, Clifford R. Jack, Sara S. Mason, Colleen S. Albers, David Knopman, Kris Johnson, William Jagust, John Q. Trojanowki, Arthur W. Toga, Laurel Beckett, Robert C. Green, Martin R. Farlow, Ann Marie Hake, Brandy R. Matthews, Jared R. Brosch, Scott Herring, Cynthia Hunt, Leslie M. Shaw, Beau Ances, John C. Morris, Maria Carroll, Mary L. Creech, Erin Franklin, Mark A. Mintun, Stacy Schneider, Angela Oliver, Jeffrey Kaye, Joseph Quinn, Lisa Silbert, Betty Lind, Raina Carter, Sara Dolen, Lon S. Schneider, Sonia Pawluczyk, Mauricio Beccera, Liberty Teodoro, Bryan M. Spann, James Brewer, Helen Vanderswag, Adam Fleisher, Pierre Tariot, Anna Burke, Nadira Trncic, Stephanie Reeder, Judith L. Heidebrink, Joanne L. Lord, Rachelle S. Doody, Javier Villanueva-Meyer, Munir Chowdhury, Susan Rountree, Mimi Dang, Yaakov Stern, Lawrence S. Honig, Karen L. Bell, Daniel Marson, Randall Griffith, David Clark, David Geldmacher, John Brockington, Erik Roberson, Marissa Natelson Love, Hillel Grossman, Effie Mitsis, Raj C. Shah, Leyla deToledo-Morrell, Ranjan Duara, Daniel Varon, Maria T. Greig, Peggy Roberts, Marilyn Albert, Chiadi Onyike, Daniel D’Agostino, Stephanie Kielb, James E. Galvin, Brittany Cerbone, Christina A. Michel, Dana M. Pogorelec, Henry Rusinek, Mony J. de Leon, Lidia Glodzik, Susan De Santi, P. Murali Doraiswamy, Jeffrey R. Petrella, Salvador Borges-Neto, Terence Z. Wong, Edward Coleman, Charles D. Smith, Greg Jicha, Peter Hardy, Partha Sinha, Elizabeth Oates, Gary Conrad, Anton P. Porsteinsson, Bonnie S. Goldstein, Kim Martin, Kelly M. Makino, M. Saleem Ismail, Connie Brand, Ruth A. Mulnard, Gaby Thai, Catherine Mc-Adams-Ortiz, Kyle Womack, Dana Mathews, Mary Quiceno, Allan I. Levey, James J. Lah, Janet S. Cellar, Jeffrey M. Burns, Russell H. Swerdlow, William M. Brooks, Liana Apostolova, Kathleen Tingus, Ellen Woo, Daniel H. S. Silverman, Po H. Lu, George Bartzokis, Neill R. Graff-Radford, Francine Parfitt, Tracy Kendall, Heather Johnson, Christopher H. van Dyck, Richard E. Carson, Martha G. MacAvoy, Pradeep Varma, Howard Chertkow, Howard Bergman, Chris Hosein, Sandra Black, Bojana Stefanovic, Curtis Caldwell, Ging-Yuek Robin Hsiung, Howard Feldman, Benita Mudge, Michele Assaly, Elizabeth Finger, Stephen Pasternack, Irina Rachisky, Dick Trost, Andrew Kertesz, Charles Bernick, Donna Munic, Marek-Marsel Mesulam, Kristine Lipowski, Sandra Weintraub, Borna Bonakdarpour, Diana Kerwin, Chuang-Kuo Wu, Nancy Johnson, Carl Sadowsky, Teresa Villena, Raymond Scott Turner, Kathleen Johnson, Brigid Reynolds, Reisa A. Sperling, Keith A. Johnson, Gad Marshall, Jerome Yesavage, Joy L. Taylor, Barton Lane, Allyson Rosen, Jared Tinklenberg, Marwan N. Sabbagh, Christine M. Belden, Sandra A. Jacobson, Sherye A. Sirrel, Neil Kowall, Ronald Killiany, Andrew E. Budson, Alexander Norbash, Patricia Lynn Johnson, Thomas O. Obisesan, Saba Wolday, Joanne Allard, Alan Lerner, Paula Ogrocki, Curtis Tatsuoka, Parianne Fatica, Evan Fletcher, Pauline Maillard, John Olichney, Charles DeCarli, Owen Carmichael, Smita Kittur, Michael Borrie, T.-Y. Lee, Rob Bartha, Sterling Johnson, Sanjay Asthana, Cynthia M. Carlsson, Steven G. Potkin, Adrian Preda, Dana Nguyen, Vernice Bates, Horacio Capote, Michelle Rainka, Douglas W. Scharre, Maria Kataki, Anahita Adeli, Earl A. Zimmerman, Dzintra Celmins, Alice D. Brown, Godfrey D. Pearlson, Karen Blank, Karen Anderson, Laura A. Flashman, Marc Seltzer, Mary L. Hynes, Robert B. Santulli, Kaycee M. Sink, Leslie Gordineer, Jeff D. Williamson, Pradeep Garg, Franklin Watkins, Brian R. Ott, Henry Querfurth, Geoffrey Tremont, Stephen Salloway, Paul Malloy, Stephen Correia, Howard J. Rosen, Bruce L. Miller, David Perry, Jacobo Mintzer, Kenneth Spicer, David Bachman, Nunzio Pomara, Raymundo Hernando, Antero Sarrael, Norman Relkin, Gloria Chaing, Michael Lin, Lisa Ravdin, Amanda Smith, Balebail Ashok Raj, Kristin Fargher

**Affiliations:** 1 College of Computer Science and Technology, Nanjing University of Aeronautics and Astronautics, Nanjing 210016, China; 2 School of Automation, Northwestern Polytechnical University, Xi’an 710072, China; 3 Department of Radiology and Imaging Sciences, School of Medicine, Indiana University, Indianapolis, IN 46202, USA; 4 School of Informatics and Computing, Indiana University, Indianapolis, IN 46202, USA; 5 Magnetic Resonance Unit at the VA Medical Center and Radiology, Medicine, Psychiatry and Neurology, University of California, San Francisco, USA; 6 San Diego School of Medicine, University of California, California, USA; 7 Mayo Clinic, Minnesota, USA; 8 Mayo Clinic, Rochester, USA; 9 University of California, Berkeley, USA; 10 University of Pennsylvania, Pennsylvania, USA; 11 University of Southern California, California, USA; 12 University of California, Davis, California, USA; 13 MPH Brigham and Women’s Hospital/Harvard Medical School, Massachusetts, USA; 14 Washington University St. Louis, Missouri, USA; 15 Oregon Health and Science University, Oregon, USA; 16 University of California–San Diego, California, USA; 17 Banner Alzheimer’s Institute, USA; 18 University of Michigan, Michigan, USA; 19 Baylor College of Medicine, Houston, State of Texas, USA; 20 Columbia University Medical Center, South Carolina, USA; 21 University of Alabama – Birmingham, Alabama, USA; 22 Mount Sinai School of Medicine, New York, USA; 23 Rush University Medical Center, Rush University, Illinois, USA; 24 Wien Center, Florida, USA; 25 Johns Hopkins University, Maryland, USA; 26 New York University, NY, USA; 27 Duke University Medical Center, North Carolina, USA; 28 University of Kentucky, Kentucky, USA; 29 University of Rochester Medical Center, NY, USA; 30 University of California, Irvine, California, USA; 31 University of Texas Southwestern Medical School, Texas, USA; 32 Emory University, Georgia, USA; 33 University of Kansas, Medical Center, Kansas, USA; 34 University of California, Los Angeles, California, USA; 35 Mayo Clinic, Jacksonville, USA; 36 Yale University School of Medicine, Connecticut, USA; 37 McGill University, Montreal-Jewish General Hospital, Canada; 38 Sunnybrook Health Sciences, Ontario, USA; 39 U.B.C. Clinic for AD & Related Disorders, Canada; 40 Cognitive Neurology - St. Joseph’s, Ontario, USA; 41 Cleveland Clinic Lou Ruvo Center for Brain Health, Ohio, USA; 42 Northwestern University, USA; 43 Premiere Research Inst (Palm Beach Neurology), USA; 44 Georgetown University Medical Center, Washington D.C, USA; 45 Brigham and Women’s Hospital, Massachusetts, USA; 46 Stanford University, California, USA; 47 Banner Sun Health Research Institute, USA; 48 Boston University, Massachusetts, USA; 49 Howard University, Washington D.C, USA; 50 Case Western Reserve University, Ohio, USA; 51 University of California, Davis – Sacramento, California, USA; 52 Neurological Care of CNY, USA; 53 Parkwood Hospital, Pennsylvania, USA; 54 University of Wisconsin, Wisconsin, USA; 55 University of California, Irvine – BIC, USA; 56 Dent Neurologic Institute, NY, USA; 57 Ohio State University, Ohio, USA; 58 Albany Medical College, NY, USA; 59 Hartford Hospital, Olin Neuropsychiatry Research Center, Connecticut, USA; 60 Dartmouth-Hitchcock Medical Center, New Hampshire, USA; 61 Wake Forest University Health Sciences, North Carolina, USA; 62 Rhode Island Hospital, state of Rhode Island, USA; 63 Butler Hospital, Providence, Rhode Island, USA; 64 University of California, San Francisco, USA; 65 Medical University South Carolina, USA; 66 Nathan Kline Institute, Orangeburg, New York, USA; 67 Cornell University, Ithaca, New York, USA; 68 USF Health Byrd Alzheimer’s Institute, University of South Florida, USA

## Abstract

Neuroimaging genetics is an emerging field that aims to identify the associations between genetic variants (e.g., single nucleotide polymorphisms (SNPs)) and quantitative traits (QTs) such as brain imaging phenotypes. In recent studies, in order to detect complex multi-SNP-multi-QT associations, bi-multivariate techniques such as various structured sparse canonical correlation analysis (SCCA) algorithms have been proposed and used in imaging genetics studies. However, associations between genetic markers and imaging QTs identified by existing bi-multivariate methods may not be all disease specific. To bridge this gap, we propose an analytical framework, based on three-way sparse canonical correlation analysis (T-SCCA), to explore the intrinsic associations among genetic markers, imaging QTs, and clinical scores of interest. We perform an empirical study using the Alzheimer’s Disease Neuroimaging Initiative (ADNI) cohort to discover the relationships among SNPs from AD risk gene *APOE*, imaging QTs extracted from structural magnetic resonance imaging scans, and cognitive and diagnostic outcomes. The proposed T-SCCA model not only outperforms the traditional SCCA method in terms of identifying strong associations, but also discovers robust outcome-relevant imaging genetic patterns, demonstrating its promise for improving disease-related mechanistic understanding.

Alzheimer’s disease (AD) is the most common form of dementia characterized by progressive impairment of memory and other cognitive functions in people over 65 years of age^1^. It is an important research topic to develop methods for early diagnosis of AD. At present, many studies have focused on searching for biomarkers from brain imaging data as well as molecular and cellular data to investigate the pathological changes^2^. To further aid the development of effective diagnostic and therapeutic approaches, it has received increasing attention to study AD at the system biology level. For example, revealing the biological pathway from the microcosmic genetic factors to macroscopic brain anatomy has the potential to understand pathogenicity mechanisms underlying the disordered cognition and behavior.

Enabled by recent advances in high-throughput genotyping and multimodal neuroimaging technologies, imaging genetics is becoming an emerging research field for discovering the associations between genetic markers such as single nucleotide polymorphisms (SNPs) and quantitative traits (QTs) extracted from structural or functional neuroimaging data^3^,^4^. Thus, it holds great promise for us to understand the complex neurogenetic and neurobiological mechanism of complex brain disorders^5^.

In prior imaging genetics studies, univariate and multivariate regression methods have been typically used to capture the effective associations between SNPs and neuroimaging data^6^,^7^,^8^,^9^,^10^. More recently, bi-multivariate analysis techniques such as various structured sparse canonical correlation analysis (SCCA) models have attracted increasing attention in brain imaging genetics to detect complex multi-SNP-multi-QT associations^11^,^12^,^13^,^14^,^15^,^16^,^17^,^18^. The essence of all the structured SCCA approaches, incorporating valuable prior knowledge, is to find the best linear transformations for imaging and genetic features respectively so that the strongest correlation between the imaging and genetic components can be achieved. These methods have the potential to discover effective imaging genetic associations, while the identified genotypic and phenotypic markers may not be disease specific. To overcome this limitation, in this paper, we focus on exploring three-way associations among genetic markers, imaging QTs, and cognitive and diagnostic outcomes. Our goal is to reveal the relationships among these multidimensional genetics, imaging and outcome data, and contribute to a better understanding of pathogenicity mechanisms in AD.

Of note, some diagnosis information guided methods have been proposed in the field of imaging genetics. The sRRR model proposed in^19^,^20^ used a two-step procedure for detecting genetic factors associated with disease relevant imaging phenotypes by using penalized linear discriminant analysis. More recently, a Bayesian framework was used to select the relevant features along the pathway from gene to imaging and then to symptom^21^. It is worth noting that both models treated diagnosis information as binary status (e.g., AD and normal control (NC)) for imaging genetic association studies. Actually, in the spectrum between NC and AD, there exist other progressive stages. For example, in the Alzheimer’s Disease Neuroimaging Initiative (ADNI) study, there were participant groups labeled as Significant Memory Concern (SMC), Early Mild Cognitive Impairment (EMCI) and Late Mild Cognitive Impairment (LMCI). In addition, the cognitive scores such as Mini-Mental State Examination (MMSE), Clinical Dementia Rating (CDR), ADNI Memory (ADNI-MEM) and ADNI Executive Functioning (ADNI-EF), which are neuropsychological assessment measures from different aspects, are often used as quantitative descriptions of symptom severity instead of binary diagnosis. Accordingly, identification of imaging genetic associations relevant to these diagnostic and cognitive outcomes may yield important information for a better understanding of disease-specific mechanisms.

With these observations, we consider the outcome-relevant imaging genetic association study as a multi-view multivariate correlation problem, which can be solved by CCA and partial least squares (PLS), as well as their sparse versions (including SCCA and SPLS)^11^,^12^,^13^,^22^,^23^. Thus, following the existing imaging genetic studies via bi-multivariate SCCA^11^, we propose a three-way sparse canonical correlation analysis (T-SCCA) framework to explore the intrinsic associations among SNP loci, neuroimaging features and clinical score outcomes. Specifically, in this study, the outcomes of interest include cognitive scores (CS) and diagnosis status (DS). We evaluate the effectiveness of the proposed method by identifying three-way associations among 85 candidate SNPs from the top AD risk gene *APOE*, 116 imaging QTs extracted from structural magnetic resonance imaging (MRI) scans, and relevant cognitive and diagnostic outcomes, using the Alzheimer’s Disease Neuroimaging Initiative (ADNI) data as a test bed. The experimental results demonstrate that the proposed T-SCCA model not only outperforms the standard two-way SCCA method in terms of identifying strong associations, but also discovers robust outcome-relevant imaging genetic patterns, demonstrating its promise for improving disease-related mechanistic understanding.

## Imaging genetic associations

### Imaging genetic associations via bi-multivariate analysis

We first describe relevant notation. We use lowercase letters to denote vectors, and uppercase letters to denote matrices. For a given matrix 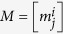, we denote its *i*-th row and *j*-th column as *m*^*i*^ and *m*_*j*_ respectively. Let *X*=[*x*_*1*_,…,*x*_*n*_]^*T*^ ∈ *R*^*n*×*p*^ be the SNP genotype data, *Y* = [*y*_*1*_, …, *y*_*n*_]^*T*^ ∈ *R*^*n*×*q*^ be the imaging QT data (i.e., voxel-based morphometry measures in this work), where *n* is the number of participants, and *p* and *q* are the number of SNPs and QTs, respectively.

For detecting complex multi-SNP-multi-QT associations, sparse canonical correlation analysis (SCCA)^11^,^12^,^13^ seeks linear transformations of variables *X* and *Y* to achieve the maximal correlation between *Xw*_*1*_ and *Yw*_*2*_ by introducing penalty terms simultaneously, which can be formulated as:


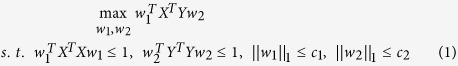


where *w*_*1*_ and *w*_*2*_ are canonical loadings or weights, reflecting the contribution of each feature in the identified canonical correlation. Note that 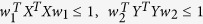 are used to embrace the covariance structure of the data in the model. 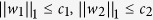 are constraints for controlling the sparsity so that only a small number of relevant features will be selected automatically from the SNP and imaging data.

### Cognitive score (CS)-relevant imaging genetic associations via three-way SCCA

In this study, for revealing the biological mechanism specific to the disease, we aim to discover imaging genetic associations that are relevant to cognitive scores (CSs) or diagnosis status. The first attempt focuses on involving multi-assessment CSs (i.e., MMSE, CDR, ADNI-MEM and ADNI-EF). Let *Z* = [*z*_*1*_, …, *z*_*n*_]^*T*^ ∈ *R*^*n*×*r*^ be the CSs, where *n* is the number of participants, *r* is the number of CSs. Since different CSs can provide complementary perspectives on neuropsychological assessments, we aim to seek a set of linear transforms to estimate the contribution of each individual cognitive score in imaging genetic associations.

The formulation of CS-relevant imaging genetic associations can be extended from eq. (1) as follows:


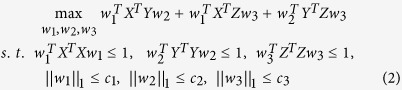


where *w*_*3*_ is the canonical loading, reflecting the contribution of each neuropsychological assessment in the identified canonical correlation. Similar to the existing constraints in eq. (1), 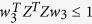 is a newly added constraint to embrace the covariance structure, and 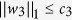 is another newly added constraint for selecting a small number of CS that are related to both imaging and genetics measures.

### Diagnosis status (DS)-relevant imaging genetic associations via three-way SCCA

Besides the CSs, the diagnosis status (DS) considered as a qualitative measurement can also describe the progressive stages of AD. Accordingly, we propose another outcome-relevant imaging genetic association that involves DS. Let *Z* ∈ *R*^*n*×1^ be the DS labels for all participants. The DS-relevant formulation, similar to eq. (2), can be used for mining DS-relevant imaging genetic associations.

It is worth noting that the equation of DS-relevant imaging genetic association is a special case of eq. (2) if *r* = 1. Similar to eq. (2), *w*_*3*_ is the canonical loading reflecting the contribution of DS in the identified canonical correlation, so that the optimization of *w*_1_,*w*_2_ and *w*_3_ can be also solved by the proposed algorithm.

## Results

### Characteristics of the dataset

The dataset comprises of 913 non-Hispanic Caucasian participants, including 211 normal control (NC), 82 significant memory concern (SMC), 273 early mild cognitive impairment (EMCI), 187 late mild cognitive impairment (LMCI) and 160 AD. In our experiments, we used the baseline structural MRI data with average voxel-based morphometry (VBM) measures in 116 regions of interest (ROIs), genotyping data with 85 candidate SNP loci, as well as cognitive scores (including MMSE, CDR, ADNI-MEM and ADNI-EF) and diagnosis status (including NC, SMC EMCI, LMCI and AD). For more details about the demographics and data processing, please see the complete information in the Methods section.

### Experimental settings

In this imaging genetic association study, 5-fold cross-validation strategy is adopted to evaluate the effectiveness of our proposed method. For parameters of regularization, we determine their values by another nested 5-fold cross-validation on the training set. It is used to fine tune the parameters in the objective function in the range of {10^−3^, 3 × 10^−3^, 10^−2^, 3 × 10^−2^, 10^−1^, 3 × 10^−1^, 1, 3, 10, 30, 100}. The parameters yielding the best performance in the inner cross-validation are finally used in the resulting model.

In the current experiments, we compare BM-SCCA (denoting conventional bi-multivariate SCCA), CS-SCCA (denoting cognitive scores-guided method via three-way SCCA), DS-SCCA (denoting diagnosis-guided method via three-way SCCA). Both CS-SCCA and DS-SCCA belong to the T-SCCA category.

### Improved association between risk SNPs and phenotypic imaging markers

We compare our proposed T-SCCA methods (including CS-SCCA and DS-SCCA) with the conventional method (BM-SCCA). The performance on each dataset is assessed using the correlation coefficient (CC) between SNP and imaging data, which is widely used for association analysis measurements. The average results of CC across the 5-fold training and testing data are calculated respectively. As shown in Table 1 and Table 2, CS-SCCA and DS-SCCA yield the CC values of 0.2633 (0.3667) and 0.2711 (0.3723) on test (training) set, respectively, which are better than those of BM-SCCA. These results indicate that the search space of BM-SCCA could be too large such that the algorithm could converge to local optima without prior knowledge, while the regularizations of the restrictions on outcome-relevant information might be able to guide imaging genetic associations out of over-fitting. This demonstrates the disease information could help improve the performances of correlations between genotypes and imaging phenotypes.

In addition, we perform a permutation test using 1000 permutations with retraining each of BM-SCCA, SC-SCCA and DS-SCCA models to assess the statistical significance of the identified correlations on the test set. The p-value corresponds to the fraction of times that the correlation coefficient is greater or equal to the result from original data. The resulting p-values (p < 0.001) are statistically significant in all three cases, including BM-SCCA, CS-SCCA and DS-SCCA, respectively.

**Figure 1:**
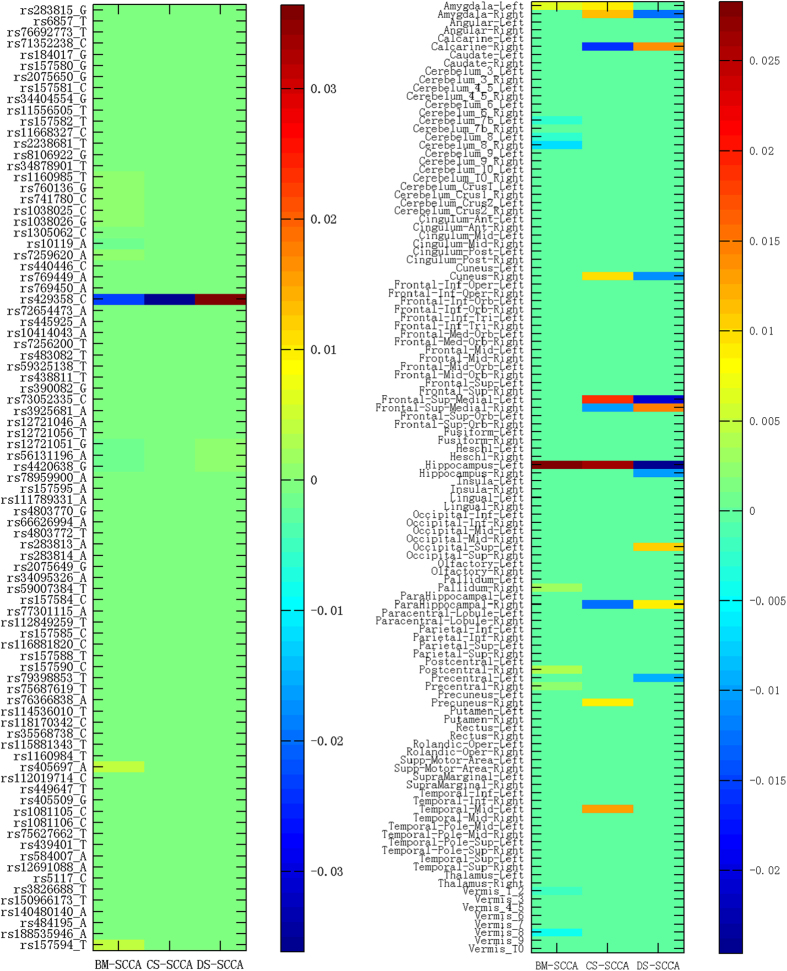
Heat map of average estimated canonical loadings on 85 *APOE* SNPs associated with 116 brain ROIs across 5-fold cross-validation respect to different methods.

Besides improving correlation performances, one major goal of this study is to identify genotypic and phenotypic markers that are not only highly correlated to each other, but also relevant to cognitive or diagnostic outcomes specific to AD. Figure 1 shows the heat map of average estimated canonical loadings on 85 *APOE* SNPs and 116 brain ROIs by BM-SCCA, CS-SCCA and DS-SCCA, respectively. The weighted colors of selected SNPs and brain regions presented by canonical loadings indicate the contributions of the corresponding genetic and phenotypic markers.

## Discussion

As expected, the well-known locus rs429358 is identified to be associated with gray matter loss in multiple AD-relevant ROIs, which is in accordance with the previous studies^24^. The C allele increases the risk of AD in *APOE* e4, which is encoded by rs429358 (www.snpedia.com/index.php/APOE) (www.alzgene.org)^25^. BM-SCCA seems to yield other loci such as rs405697 (this genetic variant in APOE region has shown only to be associated with human longevity in the literature^26^) and rs157594.

**Figure 2:**
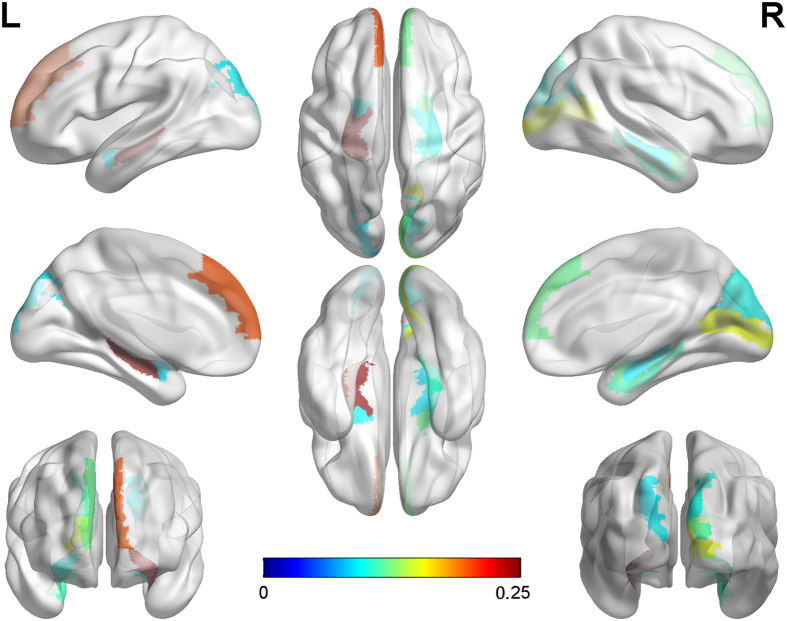
Visualization of mapping top 10 average estimated canonical loadings generated by T-SCCA (combination of CS-SCCA and DS-SCCA) onto the brain.

For the phenotype identifications, besides the well-known AD-related ROI left hippocampus, signals in the cerebellum and vermis areas are detected by BM-SCCA. However, the morphometric changes of the cerebellum have not been widely validated as AD biomarkers. On the other hand, a few additional ROIs such as right amygdala, right calcarine, right cuneus, left and right frontal-sup-medial gyrus, right parahippocampal gurus have been detected as top 10 features associated with the risk genotype biomarker rs429358 by the proposed T-SCCA (including CS-SCCA and DS-SCCA). It’s worth noting that CS-SCCA captures similar patterns on brain imaging as DS-SCCA does for the most part. More interestingly, the same ROIs selected simultaneously by two types of T-SCCA have similar weight values, which demonstrates the robust and consistent biomarker findings. Note that these weights have different signs, which are caused by the negative directionality of the cognitive score values in relation to the diagnosis values. The top 10 selected MRI-VBM imaging features, as well as their averaged estimated canonical loadings generated by T-SCCA (combination of SC-SCCA and DS-SCCA) across 5 cross-validation trials, are visualized in Fig. 2 by mapping them onto the human brain. The colors of the selected brain regions indicate the canonical loadings of the corresponding markers. The identified regions have potential clinical correlates in typical clinically well-described AD impairments. To our knowledge, the hippocampus is one of the first regions of the brain to suffer damage including memory loss and disorientation. In addition, amygdala atrophy is related to aberrant motor behavior, with potential relationships to anxiety and irritability^24^. Some existing results suggest that the magnitude of amygdala atrophy is comparable to that of the hippocampus in the earliest clinical stages of AD^27^. The analytical result reassures that our method identifies a well-known correlation between genotypes and phenotypes that is severely and consistently affected by pathology in AD. Besides confirming the prior findings, our method also yields the associations between *APOE* rs429538 and other eminent AD markers such as both left and right frontal-sup-medial gyrus. There also appear to be specific relationships among genotypes, phenotypes and neuropsychiatric symptoms that deserve further investigation.

As mentioned earlier, the quantitative CSs (MMSE, CDR, ADNI-MEM and ADNI-EF) used to index cognitive decline for disease severity are able to show the graded differences in participants, and we induce the sparsity constraint for selecting the related CSs in this imaging genetic associations study. The CS-SCCA yields the loading values of 0.0095, −0.0103, 0.0238, and −0.0026 on MMSE, CDR, ADNI-MEM and ADNI-EF, respectively. It demonstrates ADNI-MEM is the top-ranked score that contributes to the multiple associations among gene, neuroimaging and cognition. Since all CSs are neuropsychological assessment measures from different aspects, the CS-SCCA provides a simple evaluating approach to investigate the relative contribution of each clinical score.

In summary, we have performed a neuroimaging genetics study for Alzheimer’s disease (AD) to explore the relationship between genetic variations in the *APOE* gene and brain ROIs measured by voxel based morphometry (VBM). Various existing sparse canonical correlation analysis (SCCA) methods are only designed for bi-multivariate analysis, and often yield suboptimal results without considering the cognitive or diagnostic outcomes. With these observations, we have investigated a three-way sparse canonical correlation analysis (T-SCCA) framework to discover the multiple associations among SNP loci, neuroimaging features and phenotypic outcomes (including cognitive scores (CSs) and diagnosis status (DS)). The experimental results performed on 913 subjects from ADNI show that our proposed T-SCCA model can substantially achieve higher correlations between genotypic and phenotypic features. Specifically, besides the improved correlation performances, CS-SCCA captures similar patterns on canonical loadings as DS-SCCA. This supports the benefit of our general T-SCCA model that can also identify some significant and robust biomarkers in imaging genetic associations, revealing disease-specific patterns on the complex mechanisms. However, there could be different mechanisms leading to three-way associations between genetics, imaging and diagnosis. For example, on top of possible pathway from genetics to imaging and then to disease, part of the genetic influences on disease might not be mediated through features captured by neuroimaging. In addition, genetics might independently affect disease susceptibility and imaging features, resulting in association between imaging and diagnosis, or there might be hidden or confounding variables that drive these associations. Thus, it warrants further investigation to reveal the underlying mechanisms related to the three-way associations discovered by our proposed T-SCCA methods.

In this initial study, where the number of samples exceeds the number of total features, the T-SCCA model can be successfully applied for association discovery coupled with feature selection. However, if the dataset has more features than samples, this ill-conditioned problem can be addressed via dimensionality reduction or regularization. In particular, when the datasets contain far more features (e.g., SNPs at the genome-wide magnitude), it will greatly increase the computational complexity and memory requirement. Note that the normal equation in the optimization contains matrix inversion operations (the time complexity is O(n^3^), where n is the number of features). Therefore, it is an interesting future topic to develop a more efficient solution for our proposed T-SCCA and to identify potential markers from high-throughput genome-wide variants and neuroimaging quantitative traits in outcome-relevant imaging genetic studies.

In addition, in this study, we have explored the imaging genetic associations within a single population of non-Hispanic Caucasians. However, the effect of population structure is another important topic, and it may affect the identifications in multivariate associations due to the potential bias introduced by multiple populations in a study. In this case, the population structure adjustment should be considered in the study.

## Methods

### Subjects

Data used in the preparation of this study were obtained from the Alzheimer’s Disease Neuroimaging Initiative (ADNI) database (adni.loni.usc.edu). The ADNI was launched in 2003 as a public-private partnership by several organizations, including the National Institute on Aging (NIA), the National Institute of Biomedical Imaging and Bioengineering (NIBIB), the Food and Drug Administration (FDA), private pharmaceutical companies, and non-profit organizations. The primary goal of ADNI has been to test whether serial magnetic resonance imaging (MRI), positron emission tomography (PET), other biological markers, and clinical and neuropsychological assessment can be combined to measure the progression of mild cognitive impairment (MCI) and early Alzheimer’s disease (AD). The study protocols were approved by the institutional review boards of all participating centers (Nanjing University of Aeronautics and Astronautics, Northwestern Polytechnical University, Indiana University and ADNI (A complete list of ADNI sites is available at http://www.adni-info.org/.)) and written informed consent was obtained from all participants or authorized representatives. All the analytical methods were performed on the de-identified ADNI data, and were determined by Indiana University Human Subjects Office as IU IRB Review Not Required. In addition, these methods were carried out in accordance with the approved guidelines.

Participants were screened and enrolled according to criteria demonstrated in ADNI study protocol (http://adni-info.org/Scientists/ADNIStudyProcedures.html#). The general inclusion/exclusion criteria of the subjects from ADNI procedures manual (http://www.adni-info.org) are briefly described as follows:

1. NC participants have no subjective or informant-based complaint of memory decline and normal cognitive performance. The MMSE scores on NC should be between 24 and 30, CDR should be 0.
2. SMC participants have subjective memory concerns as assessed using the Cognitive Change Index (CCI; total score from first 12 items >16), no informant-based complaint of memory impairment or decline, and normal cognitive performance on the Wechsler Logical Memory Delayed Recall (LM-delayed) and the MMSE^28^.
3. EMCI participants have a memory concern reported by the subject, informant, clinician, abnormal memory function approximately 1 standard deviation below normative performance adjusted for education level on the LM-delayed, an MMSE total score greater than 24.
4. Besides a subjective memory concern as reported by subject, study partner or clinician, CDR on LMCI subjects is 0.5 and Memory Box (MB) score must be at least 0.5.
5. MMSE score on AD should be between 20 and 26, and CDR should be 0.5 or 1.0.

In the practical diagnosis of AD, multiple clinical variables are generally acquired, e.g., MMSE, CDR, ADNI-MEM (composite score for memory) and ADNI-EF (composite score for executive functioning), etc. Specifically, MMSE is used to examine functions including registration, attention and calculation, recall, language, ability to follow simple commands and orientation^29^,^30^. CDR is a numeric scale used to assess a patient’s cognitive and functional performance in six areas: memory, orientation, problem solving, community affairs, hobbies and personal care^31^. There are two derived composite scores for MEM and EF from ADNI. The formation of ADNI-MEM is complicated by the use of different word lists in the Rey Auditory Verbal Learning Test (RAVLT) and the ADAS-Cog, and by Logical Memory I data missing by design^32^. The formation of ADNI-EF includes Category Fluency-animals, Category Fluency-vegetables, Trails A and B, Digit span backwards, WAIS-R Digit Symbol Substitution, and 5 Clock Drawing items (circle, symbol, numbers, hands, time)^33^. The demographic information is summarized in Table 3.

### SNP genotype data

Since genetic risk factors can help scientists focus on relevant biological pathways and form effective hypothesis for drug design, identifying risk genetic markers associated with brain imaging can help understand the underlying biological mechanisms. We downloaded the ADNI-GO/2 genotyping data, and performed quality control and population stratification using the approach described in the previous study^34^. To limit potential effects of population stratification, this study is focused only on analyzing non-Hispanic white participants. As the best-known genetic risk factor in AD, *APOE* (located on chromosome 19) has a key role in coordinating the mobilization and redistribution of cholesterol, phospholipids, and fatty acids, and it is implicated in mechanisms such as neuronal development, brain plasticity, and repair functions^35^. Thus, we focused our analysis on all SNPs within ±20 k base pairs of the *APOE* gene boundary based on the ANNOVAR (http://annovar.openbioinformatics.org) annotation, which include a total number of 85 SNPs as candidates. For the input in the models, each SNP value was coded in an additive fashion as 0, 1 or 2, indicating the number of minor alleles.

### Imaging phenotype data

The MRI data used in this paper were also obtained from the ADNI database. We aligned the preprocessed imaging data (i.e., voxel based morphometry (VBM)) to each participant’s same visit scan, and then created normalized gray matter density maps from the MRI data in the standard Montreal Neurological Institute (MNI) space as 2 × 2 × 2 mm^3^ voxels SPM software package^36^. 116 ROI level measurements of mean gray matter densities were further extracted based on the MarsBaR AAL atlas^37^. All measurements were pre-adjusted for age, gender, and education.

### Objective function and algorithm design

In this section, we design an algorithm to solve the optimization problem defined in eq. (2). For the general formulation, using the Lagrange multiplier and writing the penalties into the matrix form, the objective function for mining CS-relevant or DS-relevant imaging genetic associations via three-way sparse canonical correlation analysis (T-SCCA) is as follows:


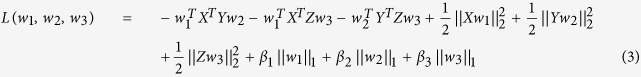


where (*β*_1_, *β*_2_, *β*_3_) and are the set of model parameters. Take the derivative regarding *w*_1_, *w*_2_ and *w*_3_ separately and let them be zero:


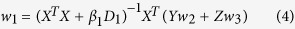



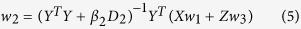



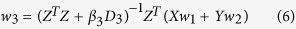


where *D*_1_ is a diagonal matrix with the *k*_1_-th element as 
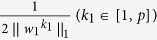
, *D*_2_ is a diagonal matrix with the *k*_2_-th element as 
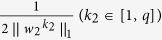
, and *D*_3_ is a diagonal matrix with the *k*_3_-th element as 
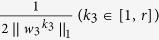
.

Since *D*_1_ relies on *w*_1_, *D*_2_ relies on *w*_2_, and *D*_3_ relies on *w*_3_, we introduce an iterative procedure to solve this objective. In each iteration, we first fix *w*_2_ and *w*_3_ to solve *w*_1_, then fix *w*_1_ and *w*_3_ to solve *w*_2_, and finally fix *w*_1_ and *w*_2_ to solve *w*_3_. The procedure stops until it satisfies a predefined stopping criterion. Algorithm 1 shows the pseudo code of the T-SCCA algorithm for mining outcome-relevant imaging genetic associations.


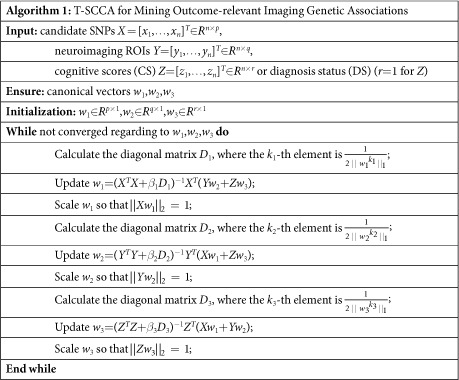


## Additional Information

**How to cite this article**: Hao, X. *et al*. Mining Outcome-relevant Brain Imaging Genetic Associations via Three-way Sparse Canonical Correlation Analysis in Alzheimer's Disease. *Sci. Rep.* **7**, 44272; doi: 10.1038/srep44272 (2017).

**Publisher's note:** Springer Nature remains neutral with regard to jurisdictional claims in published maps and institutional affiliations.

## Figures and Tables

**Table 1:** 5-fold cross-validation results on ADNI: The model learned from the training data is used to estimate the correlation coefficients on the training set.

Method	Correlation Coefficient on Training Set					
	F1	F2	F3	F4	F5	Mean + Std
BM-SCCA	0.2619	0.2810	0.1846	0.2679	0.2755	0.2542 ± 0.0396
CS-SCCA	0.3436	0.3819	0.3743	0.3536	0.3798	0.3667 ± 0.0171
DS-SCCA	0.3519	0.3843	0.3848	0.3584	0.3822	0.3723 ± 0.0159

**Table 2:** 5-fold cross-validation results on ADNI: The model learned from the training data is used to estimate the correlation coefficients on the testing set.

Method	Correlation Coefficient on Test Set					
	F1	F2	F3	F4	F5	Mean + Std
BM-SCCA	0.1996	0.1848	−0.0250	0.2845	0.2320	0.1752 ± 0.1183
CS-SCCA	0.3328	0.2126	0.2258	0.3275	0.2180	0.2633 ± 0.0612
DS-SCCA	0.3566	0.2173	0.2200	0.3139	0.2474	0.2711 ± 0.0616

**Table 3:** Characteristics of the subjects.

Subjects	NC	SMC	EMCI	LMCI	AD
Number	211	82	273	187	160
Gender(M/F)	109/102	33/49	153/120	108/79	95/65
Age	76.14 ± 6.53	72.45 ± 5.67	71.48 ± 7.12	73.86 ± 8.44	75.18 ± 7.88
Education	16.45 ± 2.62	16.78 ± 2.67	16.08 ± 2.62	16.38 ± 2.81	15.86 ± 2.75
MMSE	29.01 ± 1.23	29.00 ± 1.22	28.38 ± 1.54	27.71 ± 1.73	24.00 ± 2.62
CDR	0.01 ± 0.07	0.00 ± 0.00	0.49 ± 0.08	0.49 ± 0.07	0.72 ± 0.27
ADNI-MEM	1.02 ± 0.58	1.12 ± 0.57	0.60 ± 0.60	0.07 ± 0.67	−0.76 ± 0.61
ADNI-EF	0.85 ± 0.69	0.73 ± 0.81	0.51 ± 0.74	0.18 ± 0.81	−0.53 ± 0.91

Note: NC = Normal Control, SMC = Significant Memory Concern, ECMI = Early Mild Cognitive Impairment, LCMI = Late Mild Cognitive Impairment, AD = Alzheimer’s disease.
